# Does the Sentinel Lymph Node Sampling Alone Improve Quality of Life in Early Cervical Cancer Management?

**DOI:** 10.3389/fsurg.2020.00031

**Published:** 2020-06-12

**Authors:** Martina Gianoni, Patrice Mathevet, Catherine Uzan, Anne Sophie Bats, Laurent Magaud, Florent Boutitie, Fabrice Lécuru

**Affiliations:** ^1^UNIL et Service de Gynécologie, CHUV, Lausanne, Switzerland; ^2^Univ Lyon, Université Claude Bernard Lyon 1, Villejuif, France; ^3^Service de chirurgie gynécologique, Institut Gustave Roussy, Villejuif, France; ^4^Service de Chirurgie et Cancérologie Gynécologique et Mammaire, 83Bvd de L'hôpital, Paris, France; ^5^Service de Chirurgie Gynécologique, Hôpital Européen Georges Pompidou, Paris, France; ^6^Hospices Civils de Lyon, Pôle Santé Publique, Service Recherche et épidémiologie Cliniques, Lyon, France; ^7^Service de Biostatistiques, Batiment 4D, CHLS, Ch. Du Grand Revoyet, Pierre-Bénite, France; ^8^Service de chirurgie et cancérologie gynécologique et mammaire, Institut Curie, Paris, France

**Keywords:** cervical cancer, pelvic lymphadenectomy, quality of life, sentinel node, surgical treatment

## Abstract

**Objective:** In this subanalysis of the prospective randomized multicenter SENTICOL 2 study, we compared the quality of life (QoL), in two arms, in association with lower-limb consequences in women with early stage cervical cancer undergoing randomized sentinel lymph node (SLN) sampling alone or SLN sampling and full pelvic lymphadenectomy.

**Methods:** 206 patients with an early stage cervical cancer and a negative SLN, were randomized. Every patient had a SLN detection based on a combination of radio-isotope (Nanocis®) and blue dye (Bleu Patenté®) injections. One hundred and One patients, the “standard” group, had complete pelvic lymphadenectomy, 105 patients, the “SLN alone” group, had SLN biopsy without lymphadenectomy. At each visit (V0: preoperative, V1: 1 month, V2: 3 months and V3: 6 months following surgery) the patients completed a Short Form Health Survey (SF36) questionnaire and another questionnaire related to leg lymphedema. SF36 scores variations (compared to the baseline values) were assessed with a standard analysis and by an evaluation of the area under the curve (AUC). Several lower-limb circumferences and signs were also determined.

**Results:** General characteristics of the patients were well–balanced between groups. Physical function and general health dimensions of the SF36 scale were significantly improved at V1 and V2 in the “SLN alone” group. Mental health was also statistically better in the “SLN alone” group at V2. Other dimensions were similar. The two groups had similar evaluation at V3. AUC of SF36 sub-scores was also in favor of the “SLN alone” arm, but the difference was not statistically significant. The analysis about the lymphedema of the legs showed a reduced (but not significant) risk in the “SLN alone” group for the top-of-thigh and the mid-thigh perimeters. Lymphedema symptoms reported by the patients were significantly less severe in the “SLN alone” group.

**Conclusion:** Our study demonstrates a trend for a better quality of life and less severe leg heaviness and leg fatigue when a full pelvic lymphadenectomy is avoided.

## Introduction

For two decades, the sentinel lymph node (SLN) technique has been validated by many studies and is, currently, an integral part in the management of melanoma, breast cancer, and vulvar cancer. This technique was first used in cervical cancer ~15 years ago (1). Since then many studies have been done and have demonstrated that a radio-isotope injection into the cervix followed by lymphoscintigraphy and an intra-operative detection using a gamma probe associated to a lymphatic blue dye detection is the most efficient method to identify the SLN with detection values ranging from 87 to 100%. Also, the metastases detection rate varies between 66 and 100% (2–4) and the negative predictive value (NPV) has been found to be between 90 and 100% (2–4).

The SLN technique has several oncologic advantages like the detection of SLN located in unusual lymphatic territories (1–7) and diagnosis of low volume disease due to ultrastaging of the SLN (7–9).

At present, the surgical treatment of early stage cervical cancer is based on a radical hysterectomy or a radical trachelectomy associated to pelvic lymphadenectomy. The SLN can help in the management of these cancers while performing frozen sections on the SLN: when it is positive, in this case the hysterectomy is abandoned and a pelvic and para-aortic lymphadenectomy is done. According to the actual ESGO (10) and National Comprehensive Cancer Network (11) guidelines a negative SLN does not allow renunciation of a pelvic radical lymph node dissection (12).

Because of lymphovascular space involvement in advanced stages avoiding SLN visualization, the SLN technique would be beneficial especially in early stage cervical cancer (2, 4).

Following the results of their prospective studies, Gortzak-Uzan et al. (13) and Darlin et al. (2) recommend that the SLN technique should become the preferred procedure in the management of early stage cervical cancer with a diameter of ≤2 cm.

It has been demonstrated that a pelvic lymphadenectomy (PLND) can cause several postoperative complications, like lymphocele (14), thromboembolic events, lower limb lymphedema (14), chronic urinary retention and sexual dysfunction (14, 15). These complications impair the quality of life (QoL) of patients for the rest of their life. The withdrawal of a radical lymphadenectomy when the SLN are negative could avoid the occurrence of these postoperative complications.

Given that the SLN technique is increasingly being used in the context of cervical cancer and that pelvic lymphadenectomy, which at present is part of the management, can lead to several complications, in this study we decided to compare the quality of life and lymphedema occurrence between women undergoing pelvic lymphadenectomy and women with SLN only (both groups having negative SLN). Current study is a secondary endpoint of a prospective randomized multicenter study named SENTICOL 2.

## Materials and Methods

Between March 2009 and July 2012, a prospective randomized multicenter study named SENTICOL 2 had been performed in 30 centers in France. The protocol has been funded by the French NCI (STIC 2008). It has been registered in the NCI trial database, the number is 01639820. The main objective of the SENTICOL 2 study was the assessment of short and medium term complications of the SLN technique alone compared to a complete pelvic lymphadenectomy. One important secondary objective was the evaluation of the impact on QoL by the two different procedures. Other secondary end points were to evaluate and compare costs and results of both techniques, assess the SLN detection rate in both arms and false negative rate in the control arm, to set off recurrence location in both strategies, to evaluate disease free survival rate at 3 years and assess therapeutic changes induced by the SLN technique.

Two hundred and sixty-seven patients with a diagnosis of early stage cervical cancer (cancer stage IA1 with lymphatic vascular space invasion (LVSI) to stage IIA1) were prospectively enrolled. The inclusion criteria are presented in the supplementary section.

Among the 267 patients enrolled, 206 were randomized, 57 were non-randomized and 4 left the study. The reasons for non-randomization were unilateral SLN detection in 21 patients (7.9%), positive frozen section examination in 15 patients (5.6%), absence of SLN detection in 11 patients (4.1%) and other reasons in 10 patients (3.7%).

The number of resected lymph nodes per patient was 3.9 in the “SLN alone” arm and 16.9 (included 3.6 SLN) in the “standard” group.

Among patients without nodal involvement on frozen section and randomized in SENTICOL 2, 12 patients in the “SLN alone” arm and 9 patients in the “SLN + lymph node dissection” arm were positive at definitive pathology. Thirteen of these patients, 4 in the “standard” and 9 in the “SLN alone” group were reoperated for completion of lymph node dissection.

In the “SLN alone” group, one patient had an ilio-pelvic lymphadenectomy, two patients had a lombo-aortic lymphadenectomy and six patients underwent both iliopelvic and lomboaortic lymphadenectomy.

In the “SLN + lymph node dissection” group, 4 patients were re-operated and all of them underwent only a lombo-aortic lymphadenectomy.

All lymphadenectomies were performed through laparoscopy. The precise approach (trans-peritoneal or retro-peritoneal) of the laparoscopic resection of the nodes is not known as this data was not included in the CRF.

All para-aortic lymph node dissections were performed until the left renal vein.

In the SENTICOL 2 protocol there were no recommendations concerning re-operation of node positive patients. The choice of re-operation or not and the type of re-operation depended on the protocols of each center.

The procedure of the study consisted first in an intra-operative SLN detection, after that, depending on frozen section evaluation, the patient was either randomized or not.

The SLN detection was based on a combination of 99mTc radio-isotope injection (Nanocis®) followed by lymphoscintigraphy and blue dye injection (Bleu Patenté®).

The identified SLN were then removed.

The 206 patients who had a negative SLN at frozen section examination or did not have any frozen section examination were randomized. One hundred and one patients were allocated to the “standard” procedure group, which consisted of a complete pelvic lymphadenectomy followed by a radical hysterectomy or trachelectomy. One hundred and five were assigned to the group “SLN alone” where, after SLN detection, only a radical hysterectomy or trachelectomy without lymphadenectomy was performed. Both groups where similar regarding carcinologic prognostic factors (Table 1). Fourteen patients, 5 in the “standard” group (5.1%) and 9 in the “SLN alone” group (9.1%), had positive SLN at definitive histology and had to be re-operated (p: NS).

**Table 1:** Randomized patient and tumor characteristics.

		**SLN group**		**Standard group**		***P*-value**
		***N***	**%**	***N***	**%**	
Age (years)						0.8052
	Mean	44.19		44.61		
	Min	22.50		29.02		
	Max	72.08		81.33		
BMI (kg/m2)						0.9171
	Mean	23.63		23.90		
	Min	16.80		14.64		
	Max	41.41		40.52		
Histologic definitive diagnosis (%)						0.6774
Squamous-cell carcinoma		68	64.8	73	72.3	
Adenocarcinoma		33.00	31.4	24	23.8	
Adenosquamous carcinoma		2	1.9	2	2.0	
Other type		2	1.9	2	2.0	
FIGO stage						0.2892
	IA1 with LVSI	7	6.7	2	2.0	
	IA2	5	4.8	6	6.0	
	IB1	90	85.7	91	91.0	
	IIA	3	2.9	1	1.0	
	Missing	0	.	1	.	
SLN detection						
	Total	410		360		
	Per patient	3.9		3.6		
Rate of adjuvant therapy		13	12	16	15.8	

*BMI, body mass index; FIGO, International federation of gynecology and obstetrics; LVSI, lymphatic vascular space invasion*.

At each visit the patients were asked to complete questionnaires, including evaluation of quality of life (SF36 questionnaire). Four visits were planned: V0, preoperative and V1, V2, V3, postoperative (1, 3, and 6 months after surgery).

Questionnaires were completed in 69.3% of patients.

The SF36 questionnaire data were entered into an online database then exported to Access software. All the statistical analyses were done using Access software. However, all the SF36 questionnaires were not obtained for each patient and some questionnaires were incomplete or not correctly completed. Then, the absent or incorrect values were calculated following the standard procedure specific to the SF36 questionnaire.

With the SF36, we did two analyses of the data. First a comparison of the scores between the two groups at each visit (V0, V1, V2, V3). We analyzed the 8 sub-scores plus the two summary scores, physical component summary and mental component summary (PCS, MCS). Secondly, we calculated the area under the curve (AUC) obtained by the post operatory score variations in relation to the baseline value (V0) and compared the values between the two groups for the PCS and MCS summary scores. The AUC allows the evaluation of the postoperative decrease of the SF36 score without influence from the preoperative values.

The data necessary for the evaluation of lower limb lymphedema were acquired at each visit and were composed by objective measurements done by the gynecologist and two questionnaires, one completed by the patient and another completed by the investigator. Some prognostic factors of lymphedema were also clinically evaluated at each visit and three questions about the patient's medical history that could influence lymphedema were asked during the first visit.

Regarding leg circumference, we did a comparison between the maximal top of tight and mid-tight circumference among the postoperative visits and the top of tight and mid-tight circumference at the inclusion visit and compared the values in the two groups.

Legs perimeters were measured (result in centimeters) in both legs at top of tight and mid-tight, knee, mid-leg and ankle at each visit by the gynecologist.

To evaluate the functional signs, we decided to calculate, for each post-operative visit (V1, V2, V3), the difference (Δx) between the visual analog scale (VAS) score at the visit (Vx) and the VAS score at inclusion (V0) (Δx = V0−Vx). We then compared the values between the “SLN alone” group and the “standard” group.

## Results

### SF36

SF36 sub-scores at the inclusion visit (V0) were similar for the two groups, except for the General Health which showed a significantly better score in the “SLN alone” group (*p*-value 0.0219).

The “SLN alone” group had better scores at the postoperative visit (V1). Especially the Physical Functioning (PF) score with a mean value of −0.50 in the “SLN alone” group and of −0.97 in the “standard” group and the General Health (GH) score with a mean value of −0.23 in the “SLN alone” group and a mean value of −0.56 in the “standard” group. P-scores were statistically significant and values were, respectively, 0.0099 and 0.0398. Bodily Pain dimension was improved in the experimental arm, but the difference was not statistically significant. Other dimensions were similar among the two groups.

At the V2 visit, Physical Functioning and General Health dimensions were again improved in the “SLN alone” arm with a mean value of −0.01 in the “SLN alone” group and −0.54 in the “standard” group, *p*-value 0.0091 for PF score and mean value of 0.01 in the “SLN alone” group and −0.37 in the “standard” group, *p*-value 0.0564 for GH score. Mental Health sub-score was also statistically significant with a *p*-value of 0.0131, mean value was −0.48 in the “SLN alone” group and −1.04 in the “standard” group. PCS had a trend to better result in the experimental arm, but difference was not significant. Other sub-scores were similar between the groups.

In the analysis of the SF36 questionnaires completed 6 months after surgery (V3) we observe, like in V0, V1, and V2 questionnaire comparisons, lower values in the “standard” group but neither sub-score nor summary score have a statistically significant difference.

For more details see Table 2 and supplementary section (Supplementary Table 1).

**Table 2:** Physical and mental component summary scores.

	**V0**			**V1**			**V2**			**V3**		
	**SLN group**	**Standard group**	***P*-value**	**SLN group**	**Standard group**	***P*-value**	**SLN group**	**Standard group**	***P*-value**	**SLN group**	**Standard group**	***P*-value**
PCS												
Mean	53.5	51.73	0.3258	41.49	39.42	0.1081	47.52	44.1	0.0736	50.48	48.67	0.5177
SD	7.34	9.38		8.92	10.01		9.07	9.96		8.49	10.19	
MCS												
Mean	41.75	39.19	0.1813	41.88	38.73	0.1275	45.75	40.5	0.0106	42.99	43.82	0.8357
SD	10.61	10.79		10.18	11.92		9.71	10.84		12.45	10.66	

*PCS, physical component summary score; MCS, mental component summary score*.

To better assess the impact on quality of life of a less invasive surgery, we analyzed the area under the curve (AUC) which allows an evaluation of the impact of the SLN alone technique on the decrease of summary scores in relation to surgery.

**Figure 1:**
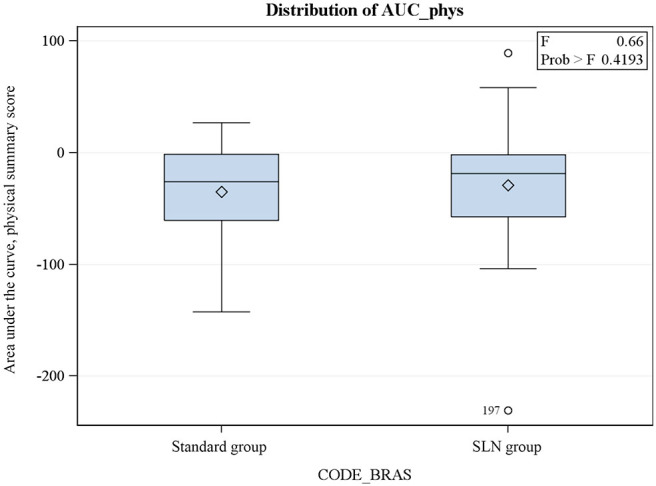
SF36 physical subscore: area under the curve (AUC) of the evolution of the score from the preoperative trough the 3 postoperative visits.

**Figure 2:**
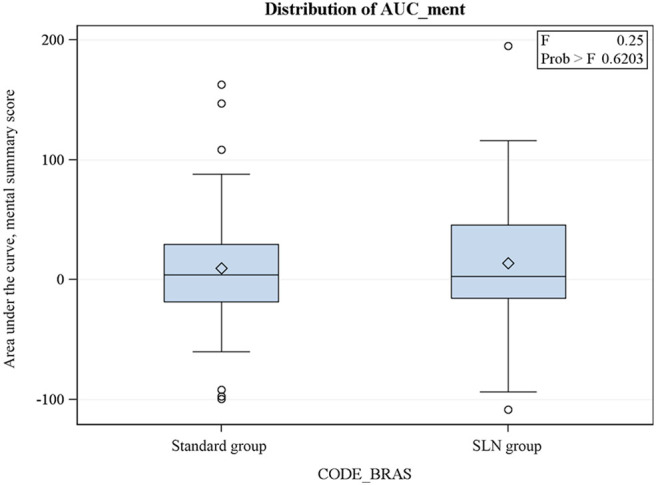
SF36 mental subscore: area under the curve (AUC) of the evolution of the score from the preoperative trough the 3 postoperative visits.

The AUC also showed a trend to better QoL in the “SLN alone” group, but the difference is not statistically significant (Figures 1, 2).

### Lymphedema

No significant difference in the risk factors of lymphedema were recorded during the different visits (data not shown).

The analyze of the lower limb perimeters measured by the investigators, showed no change in the values of knee, mid-leg and ankle circumferences at V0, V1, V2, and V3. Similarly, there was no difference between right and left side (data not shown). On the other hand, we observed a tendency to better results in the “SLN alone” group vs. the “standard” group in top of thigh and mid-thigh perimeters. However, these differences are not statistically significant (see Table 3).

**Table 3:** Percentage of increase of the circumferences of mid-thigh and top of thigh between the two groups (SLN alone vs. standard).

**Mean differential (%)**	**SLN group**	**Standard group**	***P*-value**
Mid-thigh, right	3.92	5.39	0.0784
Mid-thigh, left (%)	3.53	5.24	0.0645
Top of thigh, right (%)	2.35	4	0.1742
Top of thigh, left (%)	2.39	3.92	0.1655

*The difference is calculated between the maximal circumference among the postoperative visits and the circumference at the inclusion visit (centimeters) for mid-thigh and top of thigh*.

Regarding the functional signs, leg heaviness was significantly less reported in the “SLN alone” group (mean 0.66) than in the standard arm (mean 1.39), *p*-value 0.0482. Similarly, leg fatigue was less reported (mean 0.20) in the SLN alone group than in the lymphadenectomy group (mean 0.96), *p*-value 0.0190 (see Supplementary Table 2).

### Discussion

The results analyzed and reported here are a sub-analysis of the prospective randomized multicenter SENTICOL 2 study, realized in France between March 2009 and July 2012. The major objective of the study was the evaluation of short term (30 days post-surgery) and medium term (6 months post-surgery) morbidity of the SLN resection alone in comparison to a complete pelvic lymphadenectomy. Our results showed that the morbidity is significantly lower when only the SLN are taken (16).

To date, few studies have investigated the complications of a complete pelvic lymphadenectomy. We found seven recent studies, four prospective (15, 17–19) and three retrospective (14, 20, 21). All the studies showed that a complete pelvic lymphadenectomy could lead to many complications, like lymphocele, leg edema, deep vein thrombosis (DVT), pulmonary embolism (PE), and that the number of complications raised when an extensive PLND was done in comparison to a limited PLND or selective lymph node sampling (15, 17, 19).

Concerning QoL, we found only two studies. They demonstrated a worse QoL in presence of leg lymphedema (20, 22). We would emphasize that these studies assessed endometrial cancer patients and that no specific data is available for cervical cancer.

With the SLN technique becoming increasingly employed in cervical cancer, it seemed useful to know whether these patients could benefit from a less invasive approach.

In the study, 14 patients had to be re-operated because of positive SLN at definitive histology. Despite the important morbidity associated with the reoperations these patients were taken into account. The per protocol (PP) and the intention to treat (ITT) values were compared and no significant difference was observed. We decided to analyze the ITT values.

To accurately evaluate the quality of life, the patients were asked to complete the SF36 questionnaires. We also recorded the most important complication of this kind of surgery (the leg lymphedema) and its subjective repercussions. We did a very complete and accurate analysis of the quality of life, which until today has not been reported in early cervical cancer.

An analysis of the characteristics of the two groups of patients could exclude any confounding factor such as body mass index (BMI), age or patients' chronic diseases.

Studies have established that several factors influence the SF36 score. Advancing in age correlates with a decline in physical functioning but it does not influence the mental health score (23), the SF36 also has an inverse correlation with the BMI (24, 25) and is influenced by social class, with lower class associated with decreased health (23). Moreover, patients affected with long-standing illness or chronic physical problems have a worse health perception (23). Furthermore, the SF36 is significantly associated with morbidity and mortality at 12 months (25). These factors should not explain our results.

First, we did a simple comparative analysis of the SF36 questionnaire values. The groups were comparable at baseline, except for the General Health score, which was significantly better in the “SLN alone” group. We have no clear explanation for this result. Randomization was performed during surgery, in case of bilateral SLN detection and negative frozen section. We do not know how this selection could be influenced by General Health (this has not been reported before). A random statistical discrepancy is the most probable explanation. At the first post operative visit we observed a decrease in the QoL in both groups because of the surgery. Quality of life was significantly better in the “SLN alone” group for Physical Functioning, General Health and Mental Health dimensions. However, the General Health result is difficult to interpret since there was an unexpected difference at baseline. The difference persisted at V2 for Physical Functioning and Mental Health sub-scores. All dimensions were similar between groups at V3.

The main analysis of the SENTICOL 2 study demonstrated that avoiding a complete pelvic lymphadenectomy lead to a decrease in post-operatory morbidity. This could explain why in the “SLN alone” group we obtained statistically significant differences in the General Health and Physical Functioning sub-scores, reflecting the lower morbidity and the lower repercussion on the activities of daily life in this group of patients. This could have had repercussions on mental health, the patients being less affected postoperatively. The other aspect that could have had an influence on the significant difference concerning the Mental Health is the idea of having had a less invasive operation, and therefore being reassured.

We then analyzed the data further calculating the area under the curve produced by the SF36 score variations in relation to the baseline values (V0) for PCS and MCS summary scores. We observed a difference between the two groups in both summary scores in favor of the “SLN alone” group but none of them was statistically significant.

The analysis concerning the lymphedema of the legs revealed a very low rate of lower leg lymphedema in both groups and demonstrated that there is always a difference between the two groups in the values of top of thigh and mid-thigh perimeters, the “SLN alone” group having lower circumferences, even if these differences are never statistically significant. This result is interesting because a lymphedema of the upper leg is not described in the literature. We obtained some significant values analyzing lower limb functional signs, showing that leg heaviness and leg fatigue were significantly worse in the “standard” group. The functional signs are associated with the lymphatic circulation in legs, which can be compromised by the surgery.

In the literature, there are very few studies evaluating the impact of a complete pelvic lymphadenectomy on the limb and we found no study evaluating limb functional signs.

As we observed in our study, normally the lymphedema of the legs decreases with time, although we found a study showing an unchanged leg edema at 1 and 5 years post surgery (14).

Strength of the study are the randomized design, the prospective recording of data. Limits are the probable lack of power since this objective was only a secondary objective.

It is important to mention that our report is a secondary objective of the SENTICOL 2 study, which was put together with the aim of evaluating the morbidity of the pelvic lymphadenectomy, and this could have contributed to the fact that many of our results are not statistically significant. Moreover, the size of the study was limited and that could also have played a role. Other factors that could have contributed to our results, is the few number of sampled lymph nodes and the fact that laparoscopic lymphadenectomy does not cause important morbidity, and this could have reduced the difference between the groups.

It should be also noticed that the SENTICOL 2 study was performed with laparoscopic approach but recent data and in particular the prospective LACC Trial (26) has demonstrated that minimal invasive surgery increase the risk of recurrence in early cervical cancer treatment. So, currently the vast majority of gynecologic centers are treating early cervical cancer with a laparotomic approach. We do not know if our results could be extrapolated to a different surgical approach.

In conclusion, a complete pelvic lymphadenectomy worsens quality of life; and a more limited lymph-node dissection, as the one realized with only sentinel node resection, may lead to less morbidity and better earlier postoperative quality of life. However, mid-term evaluation (6 months postoperatively) shows no improvement in quality of life in the “SLN alone” group. In addition, long-term evaluation of quality of life and leg lymphedema in both groups of patients would be interesting to be studied in the future in order to detect and to compare late post-operative complications.

## Data Availability Statement

The raw data supporting the conclusions of this article will be made available by the authors, without undue reservation.

## Ethics Statement

The studies involving human participants were reviewed and approved by ethics committee: Comité de Protection des Personnes Lyon IV. The patients/participants provided their written informed consent to participate in this study.

## Author Contributions

MG analyzed the data and wrote the article. PM is the principal investigator of the study. He participated in the analysis of data and writing of the article. CU participated at the recruitment of the patients and analysis of data. LM participated at the study design and follow-up and at the analysis of the data. FB designed the study and did the statistical analysis of data. FL was the scientific coordinator of the study, he also participated in the analysis of data. All authors contributed to the article and approved the submitted version.

## Conflict of Interest

The authors declare that the research was conducted in the absence of any commercial or financial relationships that could be construed as a potential conflict of interest.
