# Supplementary material for: Does the Sentinel Lymph Node Sampling Alone Improve Quality of Life in Early Cervical Cancer Management?
Source: Front Surg. 2020 Jun 12;7:31. doi: 10.3389/fsurg.2020.00031 (PMC7303263; doi:10.3389/fsurg.2020.00031)
Supplement: Supplementary file 1 [file Data_Sheet_1.docx]

Supplementary Material

# Supplementary Data

On behalf of the SENTICOL 2 group:

Dr UZAN C. - l’Institut Gustave Roussy – VILLEJUIF

Dr STOECKLE E.- l’institut Bergonié  - BORDEAUX

Dr FOURCHOTTE V. - l’institut Curie – PARIS

Pr QUERLEU D.- L’institut Claudius Régaud – TOULOUSE

Dr BARON - le centre Henri Becquerel – ROUEN

Dr OTT- l’hôpital du Hasenrain – MULHOUSE

Pr DARAI E. - l’hôpital de Tenon – PARIS

Pr LEVEQUE J. - l’hôpital Sud Anne de Bretagne  - RENNES

Dr LANVIN- la clinique de l’Espérance - MOUGINS

Dr POMEL C. - le centre Jean Perrin – CLERMONT FERRAND

Pr MARRET H. - l’hôpital Bretonneau – TOURS

Dr LEBLANC E. - Le centre Oscar Lambret – LILLE

Pr HOUVENAEGHEL G.- L’institut  Paoli-Calmettes – MARSEIILLE

Pr ROUANET P. - le centre Val d’Aurelle – Paul Lamarque – MONTPELLIER

Pr DESCAMPS P. - le CHU d’ANGERS

Pr MAGE G. - le CHRU de CLERMONT FERRAND

Dr GRAESSLIN O. - l’Institut Mère Enfant - REIMS

Pr BALDAUF JJ. - l’hôpital de Haute Pierre – STRASBOURG

Pr CLASSE JM. - le centre René Gauducheau – NANTES

Pr RAUDRANT D. - le centre hospitalier Lyon Sud – LYON

Dr CONRI V. - l’hôpital Pellegrin – BORDEAUX

Pr DOUVIER S. - le CHU de DIJON

Pr BOULANGER- l’hôpital Lariboisière – PARIS

Pr LEGUEVAQUE- l’Hôpital Rangueil – TOULOUSE

Dr FOUCHE- le centre Antoine Lacassagne - NICE

Dr BOULANGER- l’hôpital Jeanne de Flandre – LILLE

Inclusion criteria

The inclusion criteria of the SENTICOL 2 study were age, absence of contraindications to laparoscopy, epithelial cervical cancer (except neuroendocrine type), cancer stage IA1 with lymphatic vascular space invasion (LVSI) to IIA1 with a diameter ≤ 4 cm, absence of pregnancy.

Patients were excluded if they had distant metastases, progressive or recurrent cervical cancer, synchronous cancer, previous pelvic lymphatic surgery, known allergy to blue dye or radioisotope, severe allergic field or if they did not understand, speak and write French.

SF36

The Short Form Health Survey with 36 questions (SF36) is a validated nonspecific and worldwide used tool for quality of life (QoL) assessment. It consist in 35 items grouped in 8 scales: Physical Functioning (PF), Role-Physical (RP), Bodily Pain (BP), General Health (GH), Vitality (VT), Social Functioning (SF), Role Emotional (RE), Mental Health (MH) plus one item who doesn’t belong to any scale. The scales are grouped in two dimensions: physical health who includes the first four scales and mental health who comprises the last four scales. Each of the 35 items is a multiple choice quantitative question. The completed questionnaires are analyzed according to a standardized procedure and three scores between 0 and 100 (total, physical health and, mental health scores), with QoL improving from 0 to 100, are obtained.

Lymphedema of the lower limbs assessment

At each visit the gynecologist measured the perimeters of the top of the thigh, the mid-thigh, the mid-leg, the knees and the ankles in both the right and the left legs. For each patient we chose the maximal perimeter measurement for top and mid-thigh among the 3 postsurgical visits and we compared it to the inclusion value for each measurement and for both right and left legs.

# Supplementary Tables

Supplementary Table 1

Subscores and summary scores results of the SF36 questionnaires in the 2 arms at the different visits

PF, Physical Functioning; RP, Role-Physical; BP, Bodily Pain, GH, General Health; VT, Vitality; SF, Social Functioning; RE, Role Emotional; MH, Mental Health; PCS, physical component summary; MCS, mental component summary

|  | V0 | | | V1 | | | V2 | | | V3 | | |
| --- | --- | --- | --- | --- | --- | --- | --- | --- | --- | --- | --- | --- |
|  | SLN group | Standard group | P-value | SLN group | Standard group | P-value | SLN group | Standard group | P-value | SLN group | Standard group | P-value |
| PF  Mean  Min  Max  P-value | 0.23  -2.60  0.68 | 0.06  -3.69  0.68 | 0.3405 | -0.50  -3.47  0.68 | -0.97  -3.47  0.68 | 0.0099 | -0.01  -2.82  0.68 | -0.54  -3.69  0.68 | 0.0091 | 0.04  -2.16  0.68 | -0.14  -3.47  0.68 | 0.3853 |
| RP  Mean  Min  Max  P-value | -0.19  -2.40  0.56 | -0.32  -2.40  0.56 | 0.5895 | -1.55  -2.40  0.56 | -1.68  -2.40  0.56 | 0.3754 | -0.75  -2.40  0.56 | -0.88  -2.40  0.56 | 0.6410 | -0.45  -2.40  0.56 | -0.57  -2.40  0.56 | 0.6429 |
| BP  Mean  Min  Max  P-value | 0.28  -1.85  1.04 | -0.01  -3.20  1.04 | 0.2428 | -0.90  -3.20  1.04 | -1.20  -3.20  1.04 | 0.0909 | -0.24  -3.20  1.04 | -0.52  -3.20  1.04 | 0.1803 | -0.07  -2.27  1.04 | -0.37  -3.20  1.04 | 0.1638 |
| GH  Mean  Min  Max  P-value | -0.08  -2.59  1.38 | -0.42  -3.33  1.38 | 0.0219 | -0.23  -2.59  1.38 | -0.56  -3.58  1.38 | 0.0398 | 0.01  -2.34  1.38 | -0.37  -3.58  1.38 | 0.0564 | 0.02  -2.34  1.38 | -0.25  -3.33  1.38 | 0.1048 |
| VT  Mean  Min  Max  P-value | -0.17  -2.93  1.87 | -0.39  -2.93  1.47 | 0.2170 | -0.77  -2.93  1.87 | -1.04  -2.93  1.87 | 0.1436 | -0.18  -2.93  1.87 | -0.58  -2.93  1.87 | 0.0639 | -0.20  -2.93  1.87 | -0.23  -2.93  1.87 | 0.8556 |
| SF  Mean  Min  Max  P-value | -0.28  -2.62  0.73 | -0.82  -3.74  0.73 | 0.0023 | -0.51  -3.18  0.73 | -0.92  -3.74  0.73 | 0.0708 | -0.30  -2.62  0.73 | -0.64  -3.74  0.73 | 0.1156 | -0.28  -3.18  0.73 | -0.54  -3.18  0.73 | 0.2003 |
| RE  Mean  Min  Max  P-value | -0.54  -2.46  0.57 | -0.60  -2.46  0.57 | 0.7635 | -1.02  -2.46  0.57 | -1.25  -2.46  0.57 | 0.2504 | -0.40  -2.46  0.57 | -0.70  -2.46  0.57 | 0.1550 | -0.39  -2.46  0.57 | -0.37  -2.46  0.57 | 0.9393 |
| MH  Mean  Min  Max  P-value | -0.81  -4.16  1.40 | -1.07  -3.46  1.05 | 0.0730 | -0.72  -3.04  0.70 | -1.23  -4.16  1.40 | 0.0071 | -0.48  -2.49  1.40 | -1.04  -3.88  0.93 | 0.0131 | -0.89  -3.60  1.40 | -0.88  -4.16  0.84 | 0.8949 |
| PCS  Mean  Min  Max  P-value | 53.50  31.60  64.65 | 51.73  24.75  66.75 | 0.3258 | 41.94  21.87  61.02 | 39.42  21.39  61.27 | 0.1081 | 47.52  28.80  62.94 | 44.10  18.08  59.86 | 0.0736 | 50.48  31.86  70.29 | 48.67  24.11  63.88 | 0.5177 |
| MCS  Mean  Min  Max  P-value | 41.75  15.04  64.12 | 39.19  13.51  63.19 | 0.1813 | 41.88  19.28  62.59 | 38.73  13.06  62.22 | 0.1275 | 45.75  20.56  65.40 | 40.50  13.40  60.64 | 0.0106 | 42.99  13.01  62.30 | 43.82  18.15  58.51 | 0.8357 |

Supplementary Table 2

Significant functional signs values between the V0 value and the maximum evolution during the 6 postoperative months. VAS = visual analogic scale.

|  |  |  | SLN group | Standard group | P-value |
| --- | --- | --- | --- | --- | --- |
| Max evolution leg fatigue / inclusion (VAS) | | |  |  |  |
|  |  |  |  |  |  |
|  |  | Mean | 0.2 | 0.96 | 0.019 |
|  |  | Min | -5.00 | -4.00 |  |
|  |  | Max | 7.00 | 8.00 |  |
|  |  |  |  |  |  |
| Max evolution leg heaviness / inclusion (VAS) |  |  |  |  |  |
|  |  |  |  |  |  |
|  |  | Mean | 0.66 | 1.39 | 0.0482 |
|  |  | Min | -5.00 | -8.00 |  |
|  |  | Max | 6.00 | 9.00 |  |
|  |  |  |  |  |  |
